# Gastric Emphysema and Hepatic Portal Vein Gas as Complications of Noninvasive Positive Pressure Ventilation

**DOI:** 10.7759/cureus.9086

**Published:** 2020-07-09

**Authors:** Harith Alataby, Mina Daniel, Joseph Bibawy, Keith Diaz, Jay Nfonoyim

**Affiliations:** 1 Internal Medicine, Richmond University Medical Center, Staten Island, USA; 2 Radiology, Richmond University Medical Center, Staten Island, USA; 3 Pulmonary and Critical Care, Richmond University Medical Center, Staten Island, USA

**Keywords:** gastric emphysema, hepatic portal vein gas, gastric vein gas, intramural gastric air, noninvasive positive pressure ventilation, nippv, bipap, emphysematous gastritis

## Abstract

Gastric emphysema (GE) in association with hepatic portal vein gas (HPVG) is a rare, benign medical condition that is very seldom caused by noninvasive positive pressure ventilation (NIPPV). This report describes a patient who developed GE along with gastric vein gas and HPVG, most likely due to multiple episodes of vomiting in combination of using bilevel positive airway pressure (BiPAP), a form of NIPPV. The patient responded to conservative treatment with intravenous fluids, pantoprazole, and the urgent cessation of BiPAP and oral intake.

## Introduction

Gastric emphysema (GE) is a rarely occurring medical condition defined as the penetration of air within the gastric wall due to a noninfectious etiology. GE is sometimes observed in association with hepatic portal vein gas (HPVG) and is generally a benign condition that can be managed conservatively; however, GE must first be distinguished from a fatal condition known as emphysematous gastritis (EG). Bilevel positive airway pressure (BiPAP) is a form of noninvasive positive pressure ventilation (NIPPV) that is rarely associated with the development of GE. Only two cases of GE secondary to NIPPV administration have been previously described. In this report, we present a case of GE with gas in the gastric and hepatic portal veins, most likely originating from NIPPV in a patient with multiple prior episodes of vomiting.

## Case presentation

A 68-year-old man with a medical history of hypertension, coronary artery disease, ischemic cardiomyopathy, heart failure with reduced ejection fraction (HFrEF), and cardiac cirrhosis was admitted to our hospital. The patient presented with difficulty breathing and mild hypoxemia secondary to acute decompensation of HFrEF.

The patient reported multiple episodes of vomiting prior to admission. The patient’s surgical history indicated the placement of numerous coronary artery stents and an automated implantable cardioverter-defibrillator. A history of periodic abdominal paracentesis secondary to cardiac cirrhosis was also reported.

On examination, the patient was tachycardic with borderline hypotension. Pulmonary examination revealed tachypnea, accessory respiratory muscle use, bilateral lung crepitations, and a blood oxygen saturation (SpO2) of 85% on a nonrebreather mask. A soft, nontender, massively distended abdomen was observed by an abdominal examination. The initial blood workup showed a white blood cell count of 12,000/µL, a hemoglobin level of 10.6 g/dL, and a platelet count of 212,000/µL. The patient had a serum potassium level of 2.7 mEq/L, a blood urea nitrogen (BUN) level of 31 mg/dL, and a creatinine level of 1.6 mg/dL. Moreover, the patient was noted to have a lactic acid level of 11 mmol/L, a thyroid-stimulating hormone level of 12 mU/L, and a pro-B-type natriuretic peptide concentration of 161,214 pg/mL. The serum ascites albumin gradient was 0.7, and liver function tests revealed mildly elevated levels.

The patient was urgently placed on NIPPV on BiPAP mode, and high-dose furosemide was administered. His clinical condition improved after a few hours, and subsequent blood work demonstrated normalized serum potassium and lactic acid levels. However, an urgent abdominal sonogram showed massive ascites and abdominal paracentesis was required due to a massively distended abdomen that was affecting the patient's respiratory effort. The blood, urine, and ascitic fluid cultures were negative for any bacterial growth.

**Figure 1:**
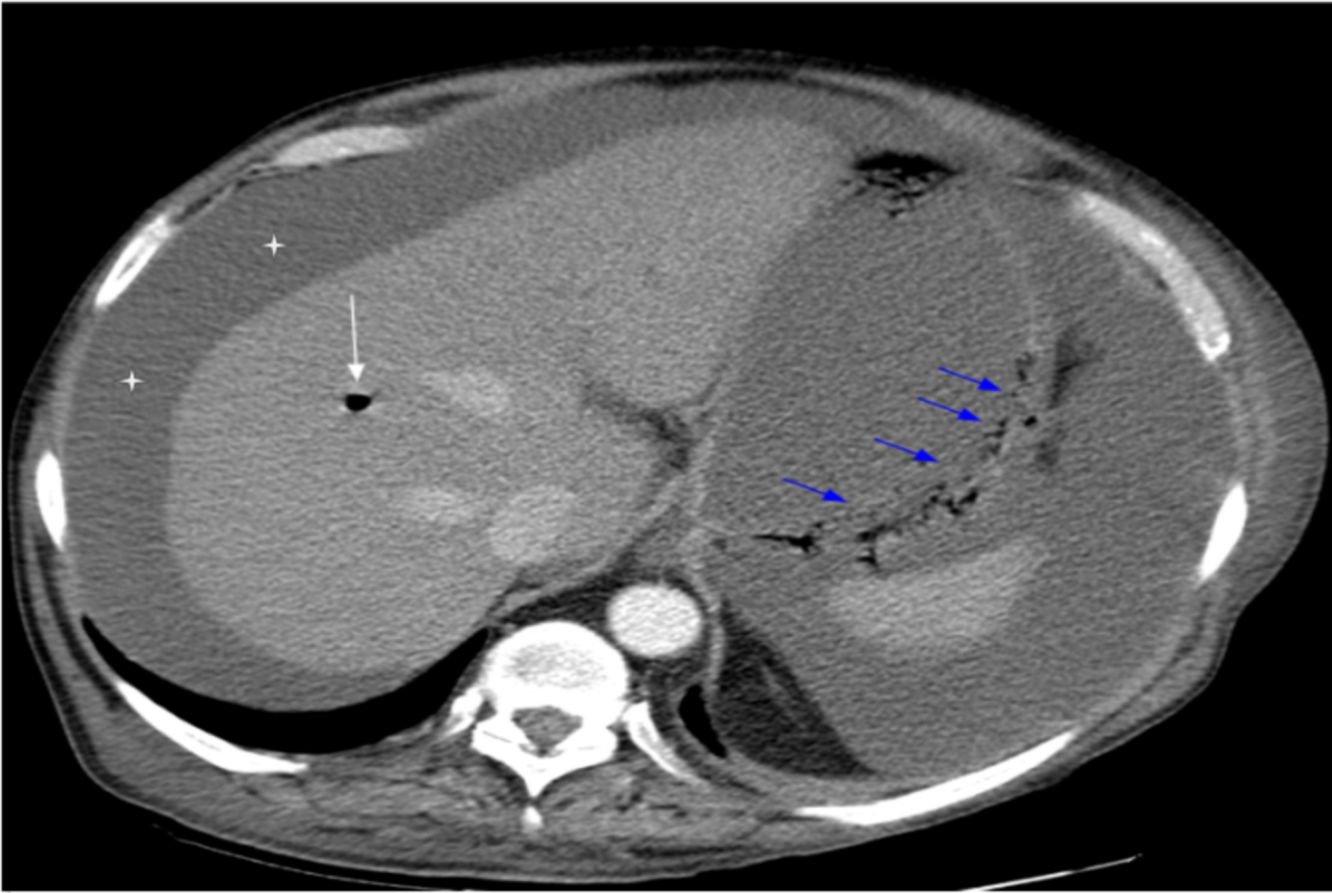
Axial images of the CT abdomen and pelvis with intravenous contrast Axial images of the CT abdomen and pelvis with intravenous contrast reveal a distended stomach with infiltration of the posterior gastric wall with air (blue arrows) consistent with emphysematous gastritis. The focus of air (white arrow) within the liver is suspicious for portal venous gas. A large amount of abdominal ascites (white stars).

**Figure 2:**
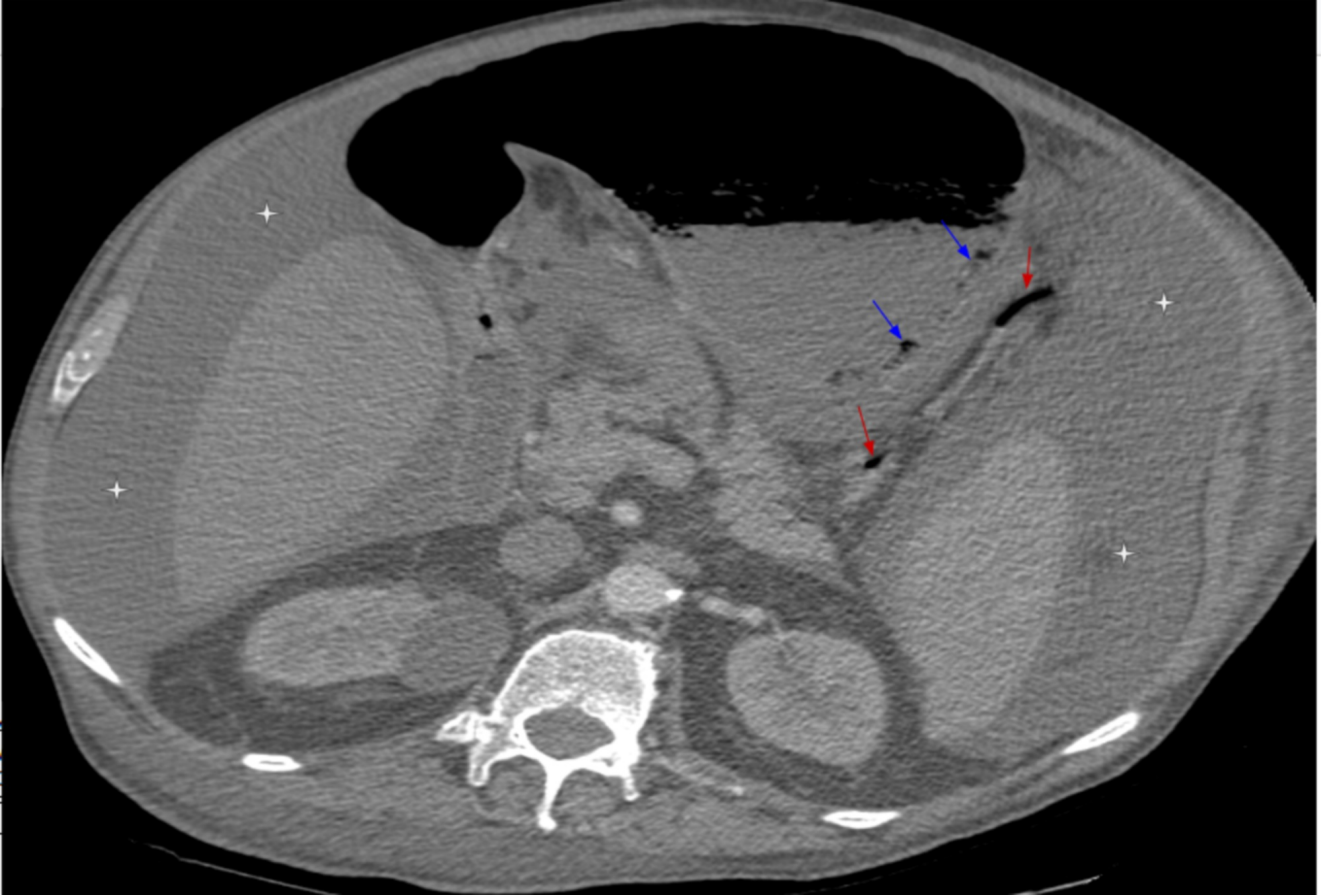
Axial images of the CT abdomen and pelvis with intravenous contrast Axial images of the CT abdomen and pelvis with intravenous contrast reveal a distended stomach with infiltration of the posterior gastric wall with air (blue arrows) consistent with emphysematous gastritis. Gastric veins were also noted to be infiltrated with air (red arrows). A large amount of abdominal ascites (white stars).

After receiving approximately 24 hours of intermittent BiPAP, the patient complained of epigastric discomfort and fullness. An abdominal CT scan with contrast showed a distended stomach and gas within the stomach wall as well as in the gastric and hepatic portal veins (Figures 1, 2).

The patient did not appear toxic and was noted to have benign findings upon abdominal examination. Additionally, his blood work did not reveal any significant anomalies. A diagnosis of GE with HPVG was established, and BiPAP and oral intake were urgently stopped for one day. Intravenous pantoprazole was administered, and the patient was placed under close monitoring.

A significant improvement in the patient’s clinical condition was observed the next day. A repeat abdominal CT scan was advised to confirm the resolution of GE but was refused by the patient, who insisted on being discharged from the hospital. The patient was discharged in stable condition, and follow-up on an outpatient basis was recommended. A repeat abdominal CT scan was performed four weeks after discharge and showed a complete resolution of gas within the gastric wall and the gastric and portal veins.

## Discussion

Pneumatosis intestinalis or intestinal pneumatosis is defined as the presence of gas within the wall of any portion of the gastrointestinal tract, from the esophagus to the rectum. Involvement of the small bowel is one of the most frequent presentations, whereas gas collection within the stomach wall is a rare finding [1,2]. EG and GE represent two relevant differential diagnoses of gas within the gastric wall.

EG, a variant of phlegmonous gastritis, is a rare and lethal infection of the stomach wall, with an overall mortality rate of approximately 60% [3]. This condition is characterized by the development of intramural gastric air secondary to infiltration of the gastric wall by gas-forming pathogenic bacteria. Clostridium perfringens, Escherichia coli, Pseudomonas aeruginosa, Klebsiella pneumoniae, and Streptococcus and Enterobacter species are the most frequently isolated organisms. Immunosuppression due to diabetes, malignancy, alcohol abuse, recent abdominal surgery, ingestion of corrosives, nonsteroidal anti-inflammatory drugs (NSAIDs), blunt abdominal trauma, and severe vomiting represent some of the risk factors that may breach the integrity of the gastric mucosa and, thus, may be associated with an increased risk of bacterial penetration into the stomach wall and the subsequent development of EG [4,5]. Severe abdominal pain, hematemesis, nausea, vomiting, epigastric tenderness, fever, chills, hemodynamic instability, leukocytosis, and lactic acidosis may be observed as presenting features [6]. CT scan of the abdomen may reveal the presence of air in the gastric wall in a streaky and linear distribution along with thickening of the gastric folds. HPVG may also be detected [7,8]. The initial treatment of EG focuses on the administration of broad-spectrum antibiotics, bowel rest, total parenteral nutrition, and serial abdominal examinations to assess for any signs of peritonitis. The role of gastrectomy during the acute phase of this condition remains unclear; however, emergency surgery should be performed in cases of gastric perforation or infarction, the involvement of a large portion of the stomach, or clinical deterioration despite optimal medical therapy. The development of gastric strictures or contractures is the most common long-term complication and may be seen in up to 25% of patients [9,10].

Unlike EG, GE is noninfectious in origin. The development of GE is generally due to a violation of the gastric mucosal integrity, followed by the forceful entry of intraluminal air into any of the layers (mucosa, submucosa, muscle layer, or serosa) of the gastric wall [11]. The air may dissect the gastric wall through various mechanisms, which can be classified into three main categories, i.e., traumatic, obstructive, or pulmonary. Traumatic GE occurs in the context of an injured gastric mucosa secondary to trauma or inflammation, where air enters the stomach wall through the disrupted mucosa. This type of GE may occur in cases of severe vomiting, esophagogastroduodenoscopy (EGD), cardiopulmonary resuscitation (CPR), acute gastric dilatation due to an eating disorder, or a penetrating gastric ulcer. Obstructive GE may develop in patients with gastric outlet obstruction, where an increased intraluminal pressure allows air to enter the gastric wall. Gastric volvulus, intestinal obstruction, gastric cancer, and hypertrophic pyloric stenosis in the pediatric population have been reported as etiological causes of obstructive GE. It has been proposed that pulmonary GE develops as air leaks from an alveolar rupture and tracks through the mediastinum, dissecting downward to enter the stomach wall [12,13].

GE typically presents with nonspecific findings. Patients are usually afebrile and may present with nausea, vomiting, mild to severe abdominal pain, epigastric discomfort, abdominal distension, or hematemesis. Most importantly, these patients do not show any signs of an acute abdomen and are almost always hemodynamically stable [14]. The diagnosis of GE is usually made radiographically. Plain abdominal radiographs may be used as an initial imaging study and may show gastric distension with linear gas shadows in the stomach wall. However, abdominal CT can detect minimal amounts of air within the gastric wall and is, therefore, the investigation of choice for diagnosing this condition. CT scan findings of GE include a hypodense lineal or curve fringe on the stomach wall along with distension. No evidence of gastric wall thickening is seen. Pneumoperitoneum, gastric vein gas, and HPVG may occasionally be detected. Endoscopic findings in GE may be nonspecific and include the presence of submucosal gas bubbles [12].

It is essential to differentiate GE from EG, as these two conditions closely resemble each other and may present a significant overlap in clinical and radiological findings, but require different management approaches. Therefore, it is important to acquire a thorough patient history and perform a comprehensive physical examination, in order to ensure that differentiating signs and symptoms are not overlooked. GE is noninfectious in origin and may arise from a number of causes, such as severe vomiting, procedural damage to the gastric mucosa during EGD, nasogastric tubes, and CPR. In contrast, EG typically arises from an infectious cause. Thus, the presence of infection supported by an elevated leukocyte count may be an important clinical feature to help distinguish EG from GE. Fever, chills, septic appearance, hemodynamic instability, and signs and symptoms of acute abdomen are also EG features that are not usually seen in patients with GE [7,9].

Bowel wall air along with HPVG may be radiologically detected in both GE and EG; however, the characteristics of the air may help differentiate these two conditions. In EG, the gastric wall air has a linear and streaky distribution pattern along with gastric fold thickening. In contrast, a hypodense lineal or curve fringe on the stomach wall arises in GE, with no evidence of gastric wall thickening. Concomitant HPVG may also be detected in both conditions, as the left gastroepiploic veins are part of the portal venous system [15].

HPVG is frequently seen with small bowel obstruction, bowel necrosis, peptic ulcer disease, mesenteric ischemia, or intra-abdominal abscess and was once considered as an indication for urgent exploratory surgery. However, several benign causes of HPVG have been identified, including GE, barium enemas, ulcerative colitis, liver transplantation, colonoscopy, and CPR. Conservative treatment without surgical intervention has been found to be successful in the management of most of these cases. Nevertheless, urgent exploratory surgery may be needed if there is a high suspicion of a necrotic or ischemic etiology [7,14-16].

We believe that in our patient, GE with gastric vein gas and HPVG was most likely caused by recurrent vomiting and BiPAP, which is a form of NIPPV. The increased gastric pressure secondary to excess positive airway pressure, along with multiple prior episodes of vomiting, may have resulted in gastric mucosal injury. The disrupted mucosa allowed the air to enter the muscular layers of the stomach and the gastric vein, and the air was then transmitted to the portohepatic system. To our knowledge, this is only the third reported case of GE and HPVG secondary to NIPPV administration.

GE typically follows a benign, acute-subacute clinical course and resolves spontaneously with conservative treatment. Once the diagnosis is established and EG has been successfully excluded, the patient can be managed with the administration of intravenous fluids and pantoprazole. In addition, the inciting factor should be identified and removed. The prognosis of GE is excellent, and complete self-resolution occurs even in the absence of any specific treatment. Long-term clinical sequelae are not generally observed, and very few cases of recurrence have been reported [12,17].

## Conclusions

GE is a rare medical condition that may be seen in association with HPVG. NIPPV is rarely associated with the development of this condition, and few case reports of NIPPV-induced GE have been described in the literature. Hence, the identification of GE with HPVG as a complication of NIPPV in a patient with prior episodes of vomiting rendered this situation as a unique case. Differentiating GE from EG is critical, as these conditions share numerous clinical and radiological features but have different management strategies and prognoses. GE is typically a benign condition and is usually treated conservatively.

## References

[1] Muhammad MN, Sadough M, King R, Singh G (2017). Pneumatosis of the esophagus and intestines with portal venous air: a rare presentation. J Community Hosp Intern Med Perspect.

[2] Treyaud MO, Duran R, Zins M, Knebel JF, Meuli RA, Schmidt S (2017). Clinical significance of pneumatosis intestinalis - correlation of MDCT-findings with treatment and outcome. Eur Radiol.

[3] Singh K (2019). Emphysematous gastritis associated with sarcina ventriculi. Case Rep Gastroenterol.

[4] Nasser H, Ivanics T, Leonard-Murali S, Shakaroun D, Woodward A (2019). Emphysematous gastritis: a case series of three patients managed conservatively. Int J Surg Case Rep.

[5] Robinson SL, Sadowski B, Eickhoff C, Mitre E, Young PE (2018). Emphysematous gastritis in a patient with untreated cyclic vomiting syndrome. ACG Case Rep J.

[6] Gupta S, Surapaneni BK, Vinayek R, Dutta SK (2018). Unexplained portal gas in a patient with an esophageal ulcer. Case Rep Gastrointest Med.

[7] Szuchmacher M, Bedford T, Sukharamwala P, Nukala M, Parikh N, Devito P (2013). Is surgical intervention avoidable in cases of emphysematous gastritis? A case presentation and literature review. Int J Surg Case Rep.

[8] Nehme F, Rowe K, Nassif I (2017). Emphysematous gastritis with hepatic portal venous gas: a shift towards conservative management. BMJ Case Rep.

[9] Iannuzzi J, Watson TJ, Litle VR (2012). Emphysematous gastritis: a young diabetic's recovery. Int J Surg Case Rep.

[10] Paul M, John S, Menon MC, Golewale NH, Weiss SL, Murthy UK (2010). Successful medical management of emphysematous gastritis with concomitant portal venous air: a case report. J Med Case Rep.

[11] Angelino V, Volpicelli G, Cardinale L (2017). Gastric emphysema after intubation. J Belg Soc Radiol.

[12] López-Medina G, Castillo Díaz de León R, Heredia-Salazar AC, Hernández-Salcedo DR (2014). Gastric emphysema a spectrum of pneumatosis intestinalis: a case report and literature review. Case Rep Gastrointest Med.

[13] Soon MS, Yen HH, Soon A, Lin OS (2005). Endoscopic ultrasonographic appearance of gastric emphysema. World J Gastroenterol.

[14] Parikh MP, Sherid M, Ganipisetti V, Gopalakrishnan V, Habib M, Tripathi M (2015). Vomiting-induced gastric emphysema and hepatoportal venous gas: a case report and review of the literature. Case Rep Med.

[15] Tang CM, Yarandi SS, Laxton WH, Khashab MA (2015). Conservative management of gastric emphysema with hepatoportal venous gas. BMJ Case Rep.

[16] Sawano T, Nemoto T, Tsubokura M, Leppold C, Ozaki A, Kato S, Kanazawa Y (2016). Asymptomatic hepatic portal venous gas with gastric emphysema as a chronic complication of gastrostomy tube placement: a case report. J Med Case Rep.

[17] Malik F, Lattanzio N, Veloso K, Nfonoyim J (2019). A case report of gastric emphysema induced by noninvasive positive airway pressure. J Community Hosp Intern Med Perspect.

